# Quantitative Susceptibility Mapping: Contrast Mechanisms and Clinical Applications

**DOI:** 10.18383/j.tom.2015.00136

**Published:** 2015-09

**Authors:** Chunlei Liu, Hongjiang Wei, Nan-Jie Gong, Matthew Cronin, Russel Dibb, Kyle Decker

**Affiliations:** 1 Brain Imaging and Analysis Center,; 2 Department of Radiology, and; 3 Center for In Vivo Microscopy, Duke University School of Medicine, Durham, NC

**Keywords:** quantitative susceptibility mapping, susceptibility tensor imaging, brain, iron, myelin, Parkinson disease, multiple sclerosis, Alzheimer disease

## Abstract

Quantitative susceptibility mapping (QSM) is a recently developed magnetic resonance imaging (MRI) technique for quantifying the spatial distribution of magnetic susceptibility within biological tissues. It first uses the frequency shift in the MRI signal to map the magnetic field profile within the tissue. The resulting field map is then used to determine the spatial distribution of the underlying magnetic susceptibility by solving an inverse problem. The solution is achieved by deconvolving the field map with a dipole field, under the assumption that the magnetic field results from a superposition of the dipole fields generated by all voxels and that each voxel has its own unique magnetic susceptibility. QSM provides an improved contrast-to-noise ratio for certain tissues and structures compared with its magnitude counterpart. More importantly, magnetic susceptibility directly reflects the molecular composition and cellular architecture of the tissue. Consequently, by quantifying magnetic susceptibility, QSM is becoming a quantitative imaging approach for characterizing normal and pathological tissue properties. This article reviews the mechanism that generates susceptibility contrast within tissues and some associated applications.

## Introduction

Quantitative susceptibility mapping (QSM) is a noninvasive magnetic resonance imaging (MRI) technique that measures the spatial distribution of magnetic susceptibility within an object (1–15). In most common practices, QSM computes the magnetic susceptibility from the phase images of gradient-recalled echoes (GREs) with the assumption that the phase shift results primarily from susceptibility-induced field inhomogeneity. This tomographic capability is unique—no other imaging techniques provide such a 3D mapping of susceptibility in the interior of an object with the measurement equipment positioned outside of the object. In imaging biological tissues and specimens, QSM has revealed a diverse range of tissue contrast in the brain and the body, reflecting the variations of tissue magnetic susceptibility (6, 8, 10, 16–24). As more tissues are being studied, the mechanisms of these contrasts are increasingly becoming more complex, which has simultaneously also allowed more potential applications in both research and clinical radiology. This article aims to review the basic mechanisms of the contrast generated by QSM and their associated applications.

## How Is QSM Generated?

**Figure 1.**
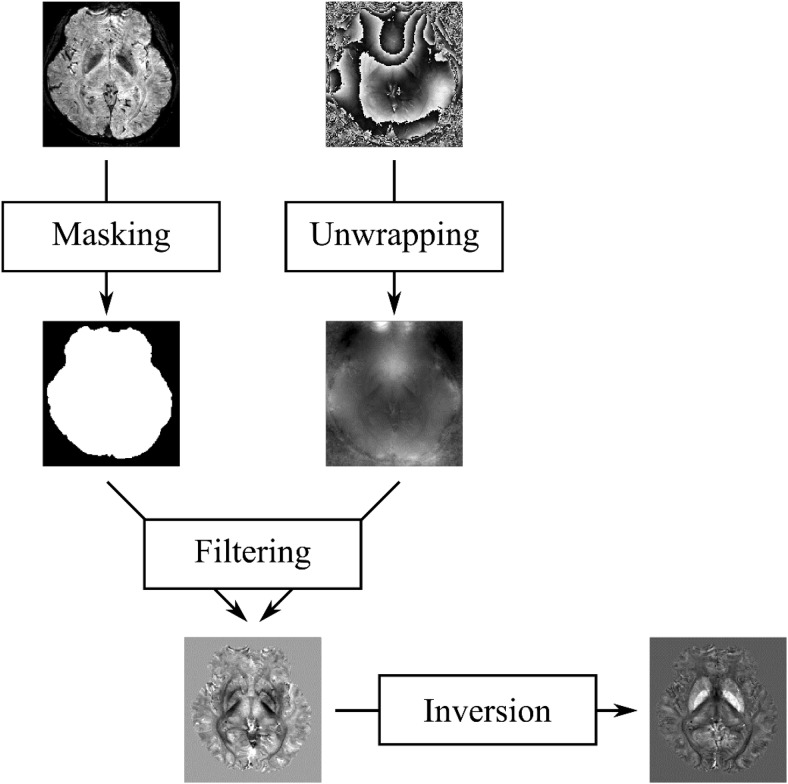
Flow chart of QSM. Magnitude and phase images are acquired with a GRE sequence. The magnitude image is used to create a mask of the brain that provides the volume of interest. The phase image is first unwrapped, and then a background phase filtering in the masked region follows. Finally, the susceptibility map is obtained by solving an inverse problem.

The field perturbations caused by inhomogeneous susceptibility within a volume of interest (VOI) may be measured from MRI phase data. GRE phase images can provide better contrast between gray and white matter in the brain than the corresponding magnitude image (25–27). However, the phase measured in GRE acquisitions is highly dependent on imaging parameters; moreover, phase values are nonlocal; ie, the phase value measured in a voxel depends not only on local tissue properties but also on the surrounding magnetic susceptibility distribution. If the susceptibility-induced magnetization is treated as a magnetic dipole, then the field perturbation caused by a known distribution of isotropic susceptibility can be obtained by convolving the susceptibility distribution with a unit dipole kernel. This calculation may be performed simply and efficiently as a pointwise multiplication in *k*-space (2, 3), such that

(1) $$\Delta B_{z}(k)=B_{0}(\frac{1}{3}-\frac{k_{z}^{2}}{\left|k^{2}\right|})\chi \left(k\right)$$

where **k** is the *k*-space vector and *k*_*z*_ its *z*-component; *B*_0_ is the applied magnetic field, taken to be in the *z*-direction; Δ*B*_*z*_ (**k**) is the Fourier transform of the *z*-component of the magnetic field perturbation; and χ (**k**) is the Fourier transform of the magnetic susceptibility distribution. QSM is achieved by inverting this equation (Figure 1). Although this inversion resolves the nonlocal property of phase, QSM faces several challenges both in the measurement of Δ*B*_*z*_ and the ill-posed nature of the inversion itself.

By scaling the measured phase by the gyromagnetic ratio and echo time (TE) to generate a field map, Δ*B*_*z*_ may be calculated from the GRE signal phase. However, it must be first ensured that the phase is indeed caused by susceptibility and not by other effects such as chemical shift, receiver-coil (**B**_1_ field) and flow-induced phases. For example, it is important to separate the phases induced by chemical shift when imaging regions of the body that have high fat content. Once the susceptibility-induced phase is isolated, the data must then be processed to remove phase wraps and background fields generated by sources outside of the VOI (Figure 1). Phase unwrapping can be easily performed using path-based (28) or Laplacian-based (8, 29) unwrapping algorithms. Background fields can be removed using a number of algorithms, including projection onto dipole fields (7, 30, 31), SHARP processing and its variants (10, 11), and the HARPERELLA algorithm (13). High-pass spatial filtering can be used to simultaneously unwrap and filter the data; however, this will also remove fields that are necessary for accurate QSM inversion. The filtered phase is then divided by the TE, yielding a map of frequency variation with respect to the reference frequency of the scanner. The local field perturbation is then given by Δ*B*_*z*_ = Δω/γ, where Δω is the local frequency perturbation and γ the gyromagnetic ratio.

Recovering a susceptibility map from a local tissue field map is more complex. The field map must be deconvolved with the unit dipole kernel corresponding to a pointwise division in *k*-space. This deconvolution is ill-posed because of zeros in the *k*-space dipole kernel on 2 conical surfaces at approximately 54.7° with respect to the direction of the main magnetic field. The inverse kernel is undefined at those surfaces, and noise is greatly amplified in regions where the kernel is very small and the inverse kernel is very large, making a simple inversion of the forward calculation impossible. In general, QSM is achieved by conditioning of the ill-posed inverse calculation to measure the susceptibility distribution while excluding or minimizing noise and artifacts.

Susceptibility maps may be calculated from a single GRE acquisition using threshold-based masking or by modifying the dipole kernel to remove or replace regions where the dipole kernel is small and the inverse kernel is very large or undefined (5, 7, 32). These algorithms are efficient and easy to implement; however, they contain severe streaking artifacts as a result of the information lost through the masking process, and a compromise must be made between noise amplification and the reduction of streaking artifacts. Streaking in the focal areas of objects with large susceptibilities such as blood vessels may be reduced by estimating the missing data using iterative (33) or compressed sensing (11) algorithms.

In addition to the conditioning of the direct inverse calculation, iterative fitting algorithms have been proposed for creating susceptibility maps by estimating the susceptibility distribution as a solution to a minimization problem. In addition to estimating the missing *k*-space data, various regularization-based optimization algorithms have been proposed using L1-norm (least absolute error) (9, 12, 20, 34) or L2-norm (least squares error) (31, 35) regularization to find a solution. Weighting based on spatial priors from the GRE magnitude (9, 36) or phase (20) may be included in the calculation to reduce streaking artifacts by enforcing smoothness in the solution in regions where the susceptibility distribution is assumed to be flat. Although these algorithms can generate good-quality susceptibility maps with minimal streaking artifacts, care must be taken on the assumptions made when selecting spatial priors to avoid reducing image contrast resulting from overregularization (9, 35, 36).

The entirety of *k*-space may be sampled using the calculation of susceptibility through a multiple-orientation sampling (COSMOS) algorithm (4). COSMOS combines data from images acquired with the region of interest oriented at multiple (≥3) angles with respect to **B**_0_. In the frame of reference of the region of interest, the dipole kernel and its ill-defined surfaces are rotated at each orientation. Appropriate selection of object orientation allows the entirety of *k*-space to be sampled, with the exception of the origin of coordinates, and a direct inversion to be performed. The advantage of this algorithm is that the complete sampling of *k*-space in the inversion process allows the recovery of the susceptibility map free of streaking artifacts. However, COSMOS is often impractical, particularly for in vivo studies, because of the additional time and potential physical difficulty in acquiring images over a range of orientations.

Although QSM has been shown by experiments to find the magnetic susceptibility distribution underlying the measured MRI signal phase with good accuracy (4, 7), its accuracy is limited by its inherent assumption that the susceptibility is isotropic in nature. In reality, some molecules such as lipids in myelin, collagen, and α-helix polypeptide (eg, in myocardial filaments) have been shown to have an anisotropic susceptibility characterized by a susceptibility tensor, creating an orientation-dependent magnetization when exposed to a magnetic field. Where such molecules form ordered structures such as myelin sheath in the brain, this can result in a measured susceptibility in QSM that varies with orientation with respect to **B**_0_. The susceptibility anisotropy within a voxel can be measured with susceptibility tensor imaging (STI) (6). STI has been used to create high-resolution fiber tracks in the mouse brain (37) and kidney (38).

## Where Does Magnetic Susceptibility Come From?

The general physical models of how materials become magnetized have become very complex given the range of natural and manmade materials that exist with diverse magnetic properties (39). Although the theory of electromagnetics is described by Maxell's equations, modeling magnetism of different materials remains a very active field of research. Much of the complexity results from the need to fully understand the collective behavior of a vast number of electrons in many different types of materials, as magnetism is believed to be predominantly contributed by the magnetic moments of electrons, with contribution from nuclear moments being negligibly small. A simple model of magnetism starting from noninteracting moments, although incomplete (eg, it does not explain the sharp transition of Curie temperature, ferromagnetism, or superconductivity), has been useful for understanding the origins of paramagnetic and diamagnetic susceptibility.

**Figure 2.**
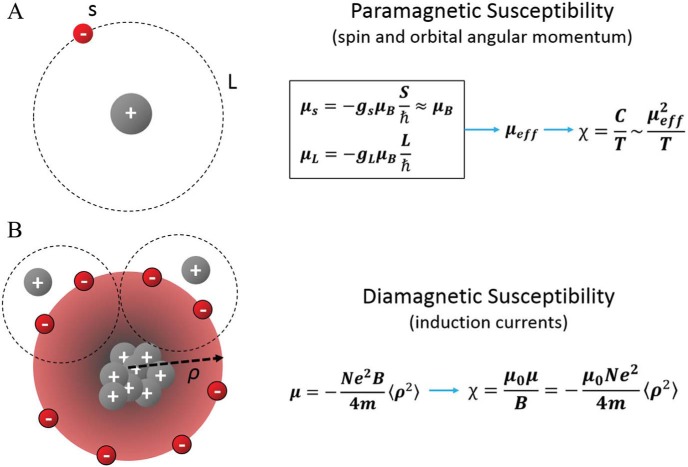
Atomic origin of paramagnetic and diamagnetic susceptibility. (A) Paramagnetic susceptibility originates primarily from spin and orbital angular momentum induced magnetic moments (μ_*s*_ and μ_*L*_, respectively) of electrons. Electrons can be found in these quantized momentum levels following the Boltzmann distribution, resulting in an expected magnetic moment μ_*eff*_ and an paramagnetic susceptibility inversely proportional to temperature. (B) Diamagnetic susceptibility originates from the precession of orbital electrons about the applied external magnetic field. The precession of electrons is modeled as a circular current, which generates a secondly field opposing the applied magnetic field. Thus, the resulting susceptibility is diamagnetic.

In an atom or molecule, electrons are distributed into different energy levels with quantized spin and orbital angular momentum, which gives rise to a set of quantized magnetic moments (39)

(2) $$\mu_{s}=-g_{s}\mu_{B}\frac{S}{\hbar }\approx \mu_{B}$$

(3) $$\mu_{L}=-g_{L}\mu_{B}\frac{L}{\hbar }$$

where μ_*s*_ and μ_*L*_ are the magnetic moment of an electron resulting from its spin and angular momentum respectively; *g* is the Landé *g*-factor; *s* and *L* are the spin and orbital angular momentum quantum number, respectively; ℏ is the reduced Planck constant; and μ_*B*_ is the Bohr magneton (Figure 2A). The probability of finding an electron with a given set of quantum numbers follows the Boltzmann's distribution, which in turn gives an effective magnetic moment μ_*eff*_. As a rule of thumb, the more unpaired electrons there are the larger the effective magnetic moment is, because paired electrons tend to cancel each other. For linear materials, magnetization (*M*) is proportional to the magnetic field (*H*), ie, *M* = χ*H* or, in a differential form, χ = ∂*M*/∂*H*. Therefore, $\chi ∼\frac{\mu_{eff}^{2}}{T}$, with the temperature coefficient being the Curie temperature *C* (Figure 2A).

In addition to the aforementioned paramagnetism, the presence of an external field also causes the electrons to precess about the applied field, generating a secondary field that opposes the applied field thus giving rise to diamagnetism. According to the Langevin theory (a classic model of nonquantum mechanics), the magnetic moment of this induced current is (39)

(4) $$\mu =-\frac{Ne^{2}\mu_{0}H}{4m_{e}}\rho^{2}$$

where *N* is the number of electrons per unit volume; *e* is the electron charge; *m*_e_ is the electron mass; μ_0_ is vacuum permeability; and ρ^2^ is the mean square distance of the electrons perpendicular to the *H* direction (Figure 2B). Therefore, the diamagnetic susceptibility is $\chi =-\frac{Ne^{2}\mu_{0}}{4m_{e}}2$, which immediately indicates that (1) this susceptibility is negative and (2) nonspherical molecules would have anisotropic magnetic susceptibility because the cross-section area is orientation-dependent.

## What Are the Factors That Influence QSM Measurements?

The susceptibility values measured by QSM are fundamentally determined by the molecular composition within an imaging voxel. They are also, however, affected by the nature of the MRI process. In bulk biological tissues as imaged by MRI, each voxel contains an ensemble of molecules of different kinds—all situated within a complex cellular environment. Given the finite resolution of MRI, susceptibility determined by QSM is only a sampled approximation of the true susceptibility distribution. This sampling process includes not only the digital sampling of the *k*-space but also the sensing of the local magnetic field based on a certain MRI signal-generating nucleus, most often the proton. As a result, the susceptibility measured by QSM is influenced by the spatial variations of proton density as well as the relaxation properties of proton spins.

### Molecular and Cellular Composition

Biological cells contain a myriad of molecules and ions. Each has its own magnetic susceptibility (40). The arrangement of cells within a tissue further complicates its magnetic property. It is thus nearly impossible to theoretically calculate the exact magnetic susceptibility of a cell or a volume of tissue. Nevertheless, within an imaged organ or body, all cells have some common features such as a lipid membrane, cytosol, and organelles, whereas QSM detects only the magnetic susceptibility variations within the tissue rather than absolute susceptibility. In other words, it is the relative portions of these molecules, especially those of strong susceptibility, that determine the contrast within a QSM image. For example, in white matter, lipids become the dominating sources as a result of the heavy myelination of axons (which is not a feature of other cell types). In deep brain nuclei, iron-containing molecules are the main sources of their paramagnetism; in the kidney, the membranes of nephrons appear to be the leading source; and in the myocardium, α-helixes of myofilaments are the major sources of anisotropy. In pathological tissues, focal depositions of minerals such as calcium, copper, and iron have been found to be a major cause of susceptibility changes.

### Tissue Microstructure

In addition to the molecular composition of a single cell, MRI-measured susceptibility is also highly dependent on the structure and arrangement of cells within a voxel. Thus, magnetic susceptibility can be used as a tool to probe tissue microstructure; for example, STI exploits the anisotropic susceptibility of certain tissues to determine the dominant orientation of the structures within a voxel, and **p-**space multipole frequency mapping aims to infer the subvoxel magnetic field distribution (41, 42).

**Figure 3.**
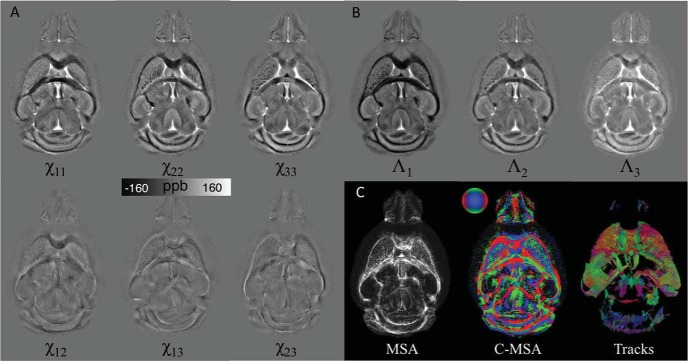
Illustration of STI on a mouse brain. Mouse brain was perfusion-fixed with formalin and stained with a gadolinium contrast agent. 3D Multiecho GRE data were acquired in 12 specimen directions on a small-bore animal 7T scanner. (A) The 6 independent tensor elements of a representative slice. (B) Λ_1_, Λ_2_, and Λ_3_ are the 3 eigenvalues representing the principal susceptibilities. (C) MSA and the color-coded MSA were calculated based on the associated eigenvector orientations. Fiber tracking was performed using Diffusion Toolkit in a process similar to DTI, and results were visualized using TrackVis. The track image shows the tracks intersecting 10 adjacent slices.

STI describes an anisotropic susceptibility tensor as opposed to the scalar quantity associated with isotropic QSM (41). Measuring the observed frequency offsets at multiple orientations with respect to the main field, *B*_0_, solves the susceptibility tensor. The susceptibility tensor is related to frequency shift in the subject frame of reference according to the following (41):

(5) $$f(k)=\gamma B_{0}\left(\frac{1}{3}\overset{\wedge }{H}\chi (k)\overset{\wedge }{H}-\overset{\wedge }{H}Ck\frac{k^{T}\chi (k)\overset{\wedge }{H}}{k^{2}}\right)$$

where χ is a second-order susceptibility tensor, **Ĥ** is the unit-applied magnetic field vector, and *B*_0_ is the magnitude of the magnetic flux density of the applied field. Assuming symmetry, there are 6 independent variables to be determined in the tensor. Thus, a minimum of 6 independent measurements are required, although fewer measurements are made possible through further assumptions and by utilizing mutual information from diffusion tensor imaging (DTI) (19, 43). Rotation of the object of interest with respect to the main field allows these independent measurements to be acquired. Estimating a susceptibility tensor is attained through inversion of the system of linear equations formed by Equation 5. The estimated susceptibility tensor can be decomposed into 3 eigenvalues, representing principal susceptibilities, and the associated eigenvectors (Figure 3). An orientation map can be formed for the principal susceptibility based on the direction of the associated eigenvector. Fiber tracks can then be reconstructed based on the STI (Figure 3) in a process similar to DTI (21, 44).

Applications of STI include but are not limited to the characterization of white matter fiber tracks both in vivo and ex vivo, as well as mapping the renal tubule and cardiac myofiber architecture ex vivo (21, 22, 41, 44).

STI of white matter is possible because of the presence of ordered bundles of axons that form fibers within the central nervous system. He and Yablonskiy pointed out that white matter frequency varies with the angle between axons and the applied magnetic field simply due to the elongated structure of the axons, but assuming isotropic susceptibility (45). Li et al. provided both theoretical and experimental data that demonstrated that the orientation-dependent susceptibility observed in the white matter results from the anisotropic susceptibility of myelin lipids. Later, Wharton and Bowtell found that when modeling axons as hollow cylinders, the anisotropic susceptibility of myelin is necessary to fully explain the observed behavior of the GRE phase (46). If sufficient multiple-orientation GRE data is acquired, the fiber tracks can be reconstructed with comparable quality to DTI in the major white matter tracks (47).

Tubular structures in the nephron of the kidney exhibit similar susceptibility anisotropy as that of white matter fibers (21). The tubules are made up of renal epithelia that also possess lipid bilayers composed of magnetically anisotropic lipid chains. For the kidney, STI may provide more extensive tracking of the tubules compared with DTI, as shown in a study on mouse kidneys (21). Whereas DTI is limited to the inner medulla, STI has the ability to track the tortuous tubules of the outer medulla and some of the cortex (21).

Significant susceptibility anisotropy is also present in the myocardium, arising from the composition and arrangement of myofilaments. A multifilament model revealed that the arrangement of diamagnetically anisotropic peptide bonds that make up the myofibers produced the bulk susceptibility anisotropy of cardiac tissue (22). Thus, STI enables mapping of the myofiber architecture, with complementary results to DTI.

Unfortunately, STI has obvious limits in terms of clinical applicability because of the multiple-orientation requirements hindered by scanning time limits and limited rotation within current transmit/receive coil arrays. Thus, nonrotational methods for investigating microstructure based on MRI-measured susceptibility are of great interest. If the structures within a voxel are heterogeneous, the magnetic field distribution within that voxel will also be heterogeneous. In principle, the field variation for a single myelinated axon will be minimal in the direction parallel to the axon but vary rapidly in the perpendicular direction. Unfortunately, the result of a standard GRE sequence is a single phase value for a given voxel that represents the summation of field offsets within that voxel. All spatial heterogeneity within the voxel is lost during the ensemble averaging. A spectral analysis technique in Fourier spectrum space, termed **p-**space, was proposed to recover the subvoxel field distribution and infer the underlying microstructure (42). This method has been proven effective through extensive simulations, but its merit under practical imaging constraints is still under investigation (42, 48–50).

### Imaging Factors

Because QSM relies on a signal-generating nucleus of an atom to sense the local field variations, the measured susceptibility is inherently influenced by the properties of these atoms. For example, are they uniformly distributed or compartmentalized? Do they have different relaxation properties? In the white matter, the myelin sheath possesses anisotropic and diamagnetic lipid chains, resulting in an increasingly more negative frequency shift in the myelin and axonal water (ie, appearing more diamagnetic) as the fiber angle increases from 0 to 90°. As the field inhomogeneity increases, the signal dephases more rapidly, which contributes to magnitude decay characterized by a varying T2* relaxation rate in GRE. The myelin water component has a short T2* compared with the axonal and extracellular space; thus, there is an absence of the myelin water signal at later TEs. An appropriate echo time must therefore be used to attain the frequency contribution from the myelin water component. Frequency shifts that originate more locally can be attained by computing the difference between a short and long TE, allowing the myelin water frequency to be isolated in a method termed frequency difference mapping (46, 51).

**Figure 4.**
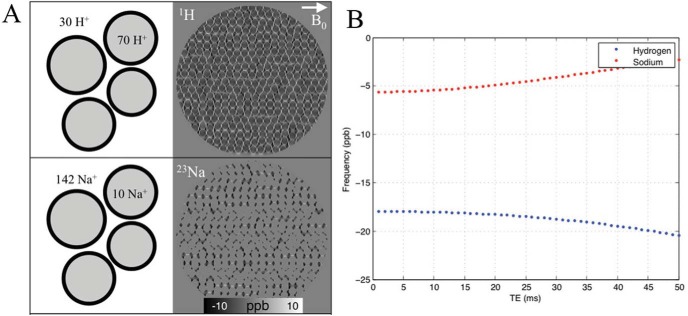
Effect of compartmentalization on measured frequency shift. (A) Simulated subvoxel frequency distribution of ^1^H and ^23^Na for a white matter fiber bundle, perpendicular to the main magnetic field at 3T. Approximately 70% of water protons are intracellular, whereas approximately 95% of ^23^Na is extracellular. (B) A single value for the voxel at each TE was attained through a complex summation of all subvoxel points. The weighting of intra-axonal and extracellular pools significantly affects the resulting frequency values and temporal evolution.

Cells also have elaborated mechanisms for maintaining the concentration gradients of many important ions and molecules across the cell membranes. For instance, ^1^H protons are more abundant in the intracellular than extracellular space (intra/extra, ∼70:30), whereas ^23^Na has a much higher concentration in the extracellular space compared with the intracellular space (extra/intra, 142:10) (34, 52). Phosphates, on the other hand, are mostly stored intracellularly (extra/intra, 2:149). Thus, in standard proton MRI, the phase values of the signal are weighted more heavily by intracellular protons, whereas in ^23^Na MRI the phase highly reflects the field contribution from the extracellular space (Figure 4).

The specific algorithmic steps taken to process the phase images and subsequently compute the susceptibility values also affect the quality of the resulting QSM maps. First, it is critical to generate a good mask of the VOI, excluding regions of unreliable phase values. Second, although different background phase removal algorithms produce phase maps that generally look similar, there are visually appreciable differences mainly in the low-frequency components. These differences may produce differences in the QSM maps, mostly around the edges. Third, the reproducibility and consistency within an inversion algorithm are generally found to be excellent (53, 54). There is still a lack of data, however, that compare different algorithms comprehensively such as truncated *k*-space division, LSQR and iLSQR, MEDI, and L1 and L2 norms (5, 8–14, 20, 35, 36).

## What Are the Clinical Applications of QSM?

Magnetic susceptibility is influenced by a wide range of physiologically significant molecules. The state and concentration of these molecules may change in diseased tissues. Therefore, QSM is being evaluated in a growing number of clinical applications. The most readily translatable applications include, among others, (1) the separation of diamagnetic calcium from paramagnetic iron, (2) the quantification of iron deposition and blood byproducts, and (3) the quantification of myelination in the white matter.

### Hemorrhage

GRE is more sensitive than computed tomography for detecting intracerebral hemorrhage (55, 56). However, T2*-weighted hypointensity in GRE suffers from blooming artifacts that are highly dependent on imaging parameters. Conversely, QSM based on GRE phase data has become sufficiently accurate for measuring the strong susceptibilities of biomaterials, including deoxyhemoglobin in the veins and blood degradation products. QSM can accurately measure the hemorrhage volumes by removing blooming artifacts inherent in traditional T2*-weighted imaging (57).

**Figure 5.**
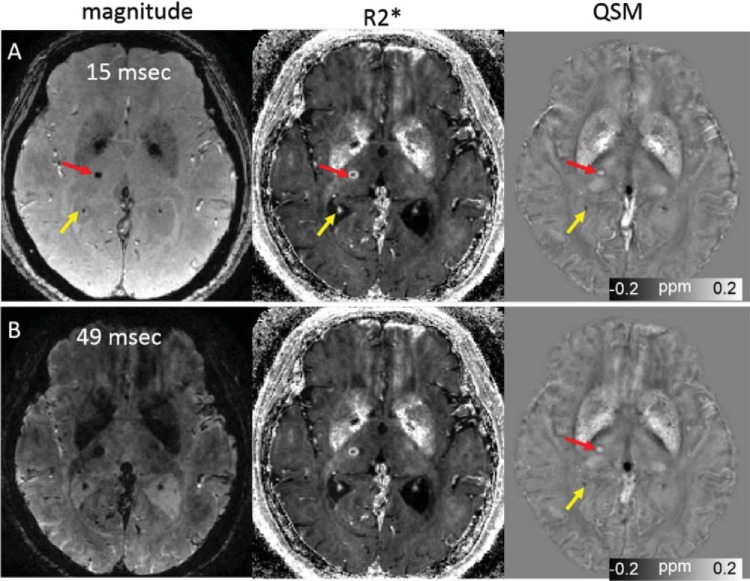
Quantitative susceptibility mapping for measuring paramagnetic (microbleeds) and diamagnetic (calcifications) biomaterials on a 45-year-old female patient. Note that calcification in the choroid plexus (yellow arrow) has a similar hyperintense appearance as a microbleed on the R2*. This ambiguity is removed on the QSM map. The scan parameters are as follows: in-plane resolution = 0.86 × 0.86 mm^2^; matrix = 256 × 256; flip angle = 12°; TE of first echo = 3 ms; echo spacing = 3.08 ms; TR = 54 ms; and number of echoes = 8. Slice thickness = 1 mm.

QSM can easily differentiate diamagnetic calcification from paramagnetic materials such as hemosiderin (58, 59), whereas both calcification and chronic hemorrhage appear hypointense on GRE magnitude images (Figure 5). Chen et al. demonstrated that QSM is superior to GRE imaging in differentiating intracranial calcifications from hemorrhage (59). Thus, QSM may be used as a more accurate detection and measurement of microbleeds in GRE MRI (60, 61). It has also been reported that QSM can reliably measure hematoma volume (8, 9). Further, during blood degradation in hemorrhage, susceptibility progressively increases from oxyhemoglobin (diamagnetic) to deoxyhemoglobin (paramagnetic), methemoglobin (strongly paramagnetic), and hemosiderin (super paramagnetic) (58, 62). Therefore, QSM may be used to precisely quantify and spatially depict dynamically evolving susceptibility over time. This susceptibility change reflects the evolution of blood product degradation thus may provide useful information allowing for a more precise management of hemorrhage patients.

### Brain Development

**Figure 6.**
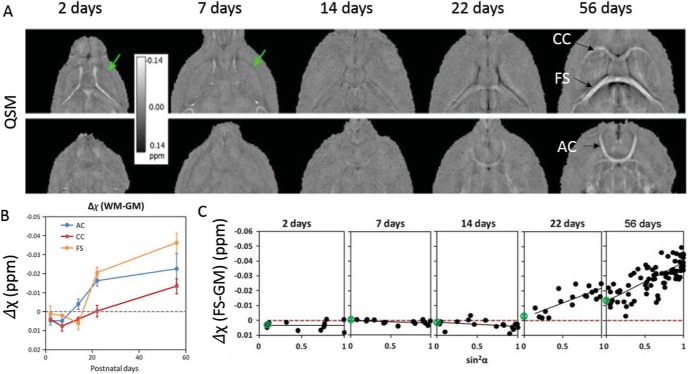
(A) Examples of magnetic susceptibility maps of the developing mouse brain. White matter largely appears paramagnetic relative to gray matter in PND2 and PND7, whereas contrast is weak at PND14. At PND22 and PND56, white matter appears to be diamagnetic. (B) Magnetic susceptibility contrast of selected white matter regions relative to neighboring gray matter as a function of age. (C) Apparent magnetic susceptibility of the fornix system as a function of sin^2^α, where α is the fiber angle with respect to **B**_0_. The slope of the fitted trend line increases as the brain develops, indicating increasing anisotropy. The scan parameters are as follows: isotropic resolution = 59 × 59 × 59 μm^3^; matrix = 368 × 184 × 184; flip angle = 40°; TE = 20 ms; and TR = 200 ms. Reproduced with permission from Argyridis et al. (66).

It is well known that the neonatal brain structure and myelination change rapidly during early development, leading to differences in brain tissue composition compared with the adult brain. Both human and mouse brains are poorly myelinated or unmyelinated at birth. Myelination occurs rapidly in the first few years of life for humans and in the first few weeks for rodents. Zhong et al. showed that phase difference between gray and white matter was greatly reduced in human neonates compared with adults (63). In the developing mouse brain from postnatal day 4 (PND4) to PND40, it was shown that phase contrast between gray and white matter correlated with the optical intensity of myelin-stained histological slides (64). However, these studies were based on phase contrast rather than the intrinsic tissue susceptibility. In the human brain, Li et al. reported that white matter became more diamagnetic as the brain developed from 1 to 83 years of age (65). In the mouse brain, Argyridis et al. (66) evaluated the temporal evolution of magnetic susceptibility in the white matter of mouse brain from PND2 to PND56. They showed that, at PND2 and PND7, white matter appears paramagnetic compared to surrounding gray matter (Figure 6). Its magnetic susceptibility then became increasingly diamagnetic as the brain developed. Furthermore, the increasing diamagnetism correlated well with the increasing myelin as depicted by myelin staining intensity.

Besides the diamagnetism, another important characteristic of white matter is that the magnetic susceptibility anisotropy (MSA) is directly proportional to myelin concentration (67). Argyridis et al. (66) also found that susceptibility anisotropy increased monotonically as a function of age from PND2 to PND56. It was further shown that MSA reached 0.02 ppm by PND22 compared to just −0.0028 ppm at PND14 and continued to grow through PND56, reaching 0.026 ppm (Figure 6).

The sensitivity of QSM to myelination may therefore be useful for monitoring delayed myelination or the loss of myelination during early brain development. For example, in a recent study of a mouse model of fetal alcohol spectral disorder, QSM revealed clear and significant abnormalities in the anterior commissure, corpus callosum, and hippocampal commissure that likely resulted from reduced myelination (68). The study also suggested that QSM may be even more sensitive than DTI for examining changes resulting from prenatal alcohol exposure.

### Aging

In white matter, the measured magnetic susceptibility has been related to diamagnetic myelin lipids and proteins of the myelin sheath. Li et al. observed a biphasic pattern of susceptibility change in white matter tracts, such as the internal capsule, the splenium of corpus callosum, and the optic radiation, with an initial decrease followed by an increase (65). This is consistent with known maturation and decay in the course of normal brain development and aging (69). The temporal characteristics, especially the time to reach minimum susceptibility, vary among different white matter fiber bundles. For instance, susceptibilities of the internal capsule, the splenium of corpus callosum, and the optic radiation reach their minimums at 45, 32, and 26 years, respectively.

In deep gray matter, iron accumulation throughout the lifespan has been well documented. Interest in quantifying iron deposition in deep gray matter regions using QSM has increased recently. Combined with x-ray fluorescence imaging, an ex vivo QSM study has established positive correlations between iron measurements and susceptibility values in deep gray matter regions (70). In 2 groups of extreme age (elderly group: age = 74.4 ± 7.6 years, n = 11; young group: age = 24.0 ± 2.5 years, n = 12), Bilgic et al. demonstrated a strong significant correlation between susceptibility and postmortem iron measurements in deep gray matter regions (*r* = 0.881). Significantly higher susceptibility values in the elderly group versus the young group have also been observed in regions such as the putamen, globus pallidus, and red nucleus (16).

In a more comprehensive study of 191 consecutively aged individuals (7-87 years), nonlinear increase of susceptibility with aging has been observed in the globus pallidus, red nucleus, substantia nigra (SN), and dental nucleus. Relatively more linear increases with aging were found in the putamen and caudate nucleus throughout all ages investigated. The plateau of susceptibility in the globus pallidus was found between the ages of 20 to 30 years. One specific finding in the globus pallidus is the inner and outer globus pallidus can be differentiated before the age of 27 years that later become indistinguishable. One possible explanation is that the medial medullary lamina grows thinner with aging. Higher susceptibility in the posterior putamen than the anterior has also been observed after the age of 27 years (65). It is important to note that elevated iron in this healthy elderly group was found to be associated with motor function decline (71).

In a recent study focused on age-related susceptibility change after the age of 20 years, Gong et al. reported that regional susceptibility levels (ranking from highest to lowest) are as follows: the globus pallidus, substantia nigra, red nucleus, caudate nucleus and putamen, and thalamus. In the age range investigated (∼25-78 years), linear age effects on susceptibility values were confirmed in regions other than the globus pallidus, and the rates varied, with the putamen exhibiting the highest rate of increase—twice that observed in the substantia nigra and caudate nucleus. Gong et al. further showed that hemisphere and gender-related differences existed in deep gray matter regions. Significant leftward asymmetries in iron content were observed in the substantia nigra and caudate nucleus. Gender difference was observed in the thalamus and red nucleus, where men have a higher iron level than women (72). These findings in deep gray matter regions may provide new clues for unveiling the underlying mechanisms of iron-related neurodegenerative diseases.

### Parkinson Disease and Presurgical Planning

The development of Parkinson disease (PD) is associated with dopaminergic cell loss and iron accumulation in the pars compacta (PC) within the SN. Recent studies have shown QSM to be a potentially useful tool in helping to diagnose and treat PD because of its sensitivity to variation in iron levels. In vivo studies have shown that QSM values are increased in the PC in PD patients relative to those measured in healthy controls (73–75) and that QSM is more sensitive than R2 and R2* measurements in discriminating between patients and healthy controls (74, 75). As such, QSM provides a useful, more quantitative means of assessing abnormal iron deposition in PD.

**Figure 7.**
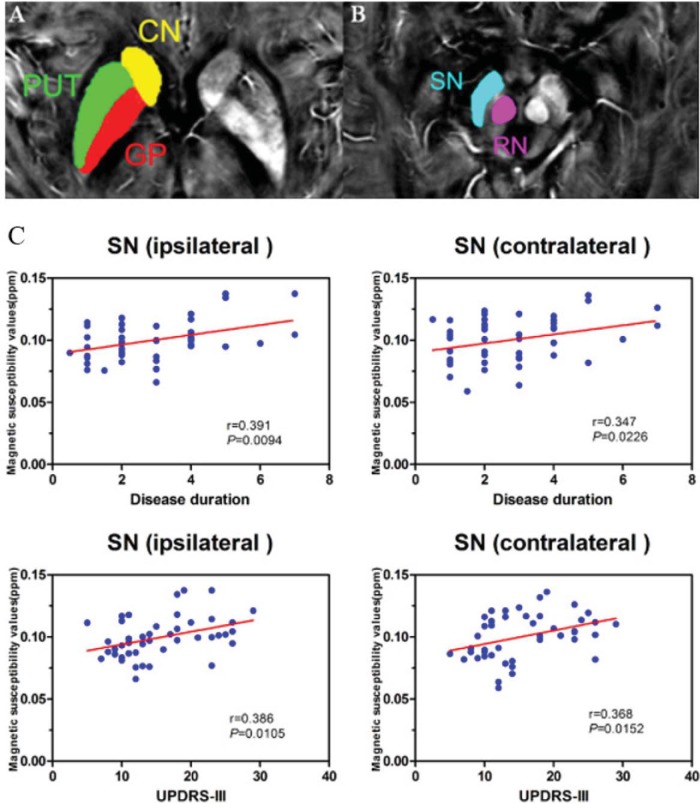
QSM of early-stage PD illustrates the regions of deep brain nuclei (A, B): the head of caudate nucleus (CN); putamen (PUT); globus pallidus (GP); substantia nigra (SN); and red nucleus (RN). (C) Scatter plots and regression lines show the significant relationship between susceptibility values in bilateral SN and clinical measures in early-stage PD. Correlations are partialed for age. The susceptibility value of ipsilateral SN is positively correlated with disease duration (top left: *r* = 0.391, *P* = .0094) and UPDRS III score (bottom left: *r* = 0.386, *P* = .0105) in PD. The susceptibility value in SN contralateral to the most affected side in PD patients is positively correlated with disease duration (top right: *r* = 0.347, *P* = .0226) and UPDRS III score (bottom right: *r* = 0.368, *P* = .0152). Reproduced with permission from He et al. (76).

QSM has also been found to be sensitive enough to detect disturbed iron distribution in early idiopathic PD. In a recent study by He et al. (76), the intergroup differences of susceptibility and R2* value in deep gray matter nuclei, including the head of caudate nucleus, putamen, global pallidus, substantia nigra, and red nucleus, and the correlations between regional iron deposition and the clinical features were explored in 44 early PD patients and 35 gender- and age-matched healthy controls. Susceptibility values were found to be elevated within the bilateral SN and red nucleus contralateral to the most affected limb in early PD compared with healthy controls. In comparison, increased R2* values were only seen within the SN contralateral to the most affected limb in the PD group compared with controls. Furthermore, it was found that bilateral SN magnetic susceptibility positively correlated with disease duration and the Unified Parkinson Disease Rating Scale III (UPDRS III) scores in early PD (Figure 7). This finding further supports the potential value of QSM as a noninvasive quantitative biomarker of early PD.

Deep brain stimulation (DBS) is an effective treatment for the symptoms of PD (77, 78) and involves implanting stimulating electrodes in the brain. Precise placement of these electrodes is essential for delivering the desired effects and minimizing side effects (79–82), and presurgical imaging is essential for determining patient suitability and guiding surgery (83–85). The dorsolateral portion of the subthalamic nucleus has been identified as the optimal stimulation site in the treatment of PD (86); the medial globus pallidus may also be targeted (87). QSM has been shown to be superior to conventional MRI protocols in depicting both the subthalamic nucleus (88) and medial globus pallidus (87), meaning that it may improve presurgical planning for treating PD with DBS.

### Multiple Sclerosis

**Figure 8.**
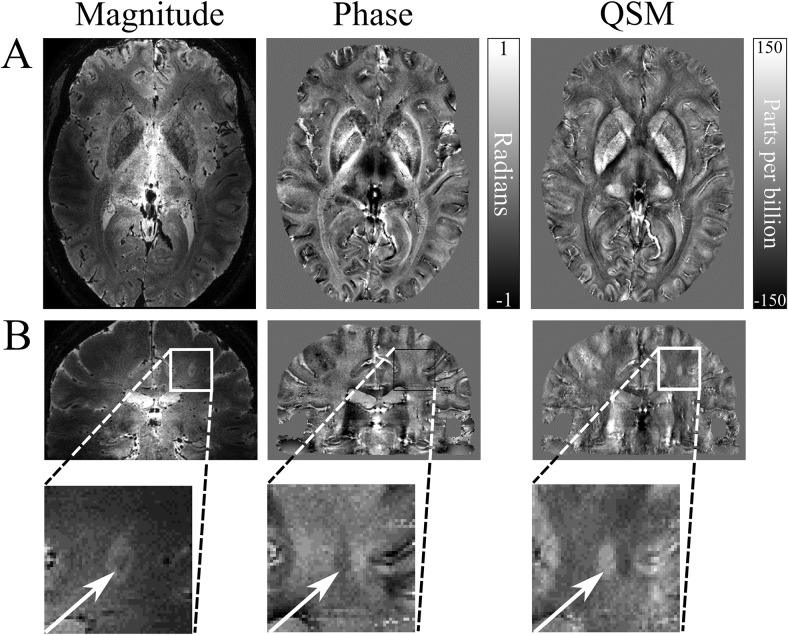
Examples of QSM in the investigation of MS. (A) QSM values can be used as a marker of iron deposition in the deep gray matter, which is characteristic of MS. The susceptibility map shows good delineation of the deep gray matter structures in closer agreement with the magnitude image than the nonlocal phase contrast. (B) Some white matter lesions identified on GRE magnitude images appear in susceptibility maps as a result of either reduced myelin content, increased iron content, or a combination of these factors. Here, the QSM data depicts the lesion with a clear hyperintense core; however, the phase contrast is distorted, with a poorly defined lesion edge. The scan parameters are as follows: 7T; 0.5-mm^3^ isotropic resolution; TE = 20 ms; TR = 150 ms; FOV = 196 × 164 × 85 mm^3^; EPI factor = 3; and SENSE factor = 2. Images courtesy of the Sir Peter Mansfield Imaging Centre, University of Nottingham, United Kingdom.

MRI is a well-established tool in diagnosing and investigating multiple sclerosis (MS); however, common MRI measurements such as lesion number or total lesion volume have not been shown to predict disease progression (89). QSM has become increasingly prominent in the search for a quantitative biomarker to measure tissue changes that occur in MS, with a focus on quantifying iron levels in the deep gray matter, and identifying regions of demyelination and iron accumulation during the formation of MS lesions (Figure 8) (17, 90–99).

Langkammer et al. (18) showed that susceptibility values in the deep gray matter measured using QSM correlated with increased iron levels measured using inductively coupled plasma mass spectrometry, suggesting that in this region QSM values may be assumed to mainly reflect local iron levels. Several studies have shown that QSM values are significantly increased in the deep gray matter in patients with clinically definite MS or a clinically isolated syndrome that suggests MS relative to healthy controls (90, 91, 95); in addition, these increases have been shown to correlate with expanded disability status scale measures of disability (95). It is thought that iron accumulation in the central nervous system may promote damage through oxidative stress, blocking repair mechanisms, activating microglia and macrophages, and/or facilitating mitochondrial changes that lead to cellular degradation (100). Combining QSM and R2* data has been shown to improve automated segmentation of deep gray matter structures in high (_3T_ and higher) field-strength MRI compared with segmentation based on T1-weighted images.

Assessing white matter changes in MS using QSM is more challenging because of the combination of demyelination, which causes a net increase in susceptibility as a result of the loss of diamagnetic myelin, and the accumulation of paramagnetic iron in ferritin and presence of iron-bearing inclusions such as microglia and macrophages, which also causes an increase in susceptibility. Furthermore, the loss of iron-bearing oligodendrocytes during demyelination may offset some myelin loss by decreasing net susceptibility (99). Although attempts have been made to separately quantify iron and myelin levels by combining QSM data with quantitative R2* data (101), recent studies of white matter lesions in MS have inferred the definite presence of iron from QSM values greater than 0 ppm relative to the ventricular cerebrospinal fluid (CSF), because complete demyelination cannot raise the susceptibility beyond this value (97).

QSM is increasingly used in the study of MS lesions, where it has been shown to more accurately depict the heterogeneous distribution of magnetic susceptibility in such lesions more accurately than phase imaging (96). Li et al. compared the appearance of MS lesions identified using MPFLAIR and T1 MPRAGE magnitude images in R2* and QSM images and found that lesions may be either isointense or hyperintense in QSM and are mostly diamagnetic relative to CSF, with QSM isointense lesions suggesting slightly higher myelin levels (99). A small number of lesions may have a positive susceptibility relative to CSF, suggesting increased iron levels and possibly complete demyelination (99). Wisnieff et al. (97) compared QSM of white matter lesions to histological iron staining and suggested that in completely demyelinated lesions, iron levels may be directly quantified using QSM. Chen et al. (94) compared the temporal evolution of QSM values in white matter lesions and found that susceptibility increased from similar values to normal-appearing white matter (NAWM) in acute enhancing lesions and retained significantly higher susceptibilities in early to intermediate nonenhancing lesions, but returned to similar values to normal-appearing white matter in chronic nonenhancing lesions.

### Alzheimer Disease

The basal ganglia are reported as the earliest and most intense accumulators of β-amyloid (Aβ) in individuals genetically predisposed to develop Alzheimer disease (AD) in the future (102). Iron overload in the basal ganglia is also a well-known feature of AD (103). Therefore, accurately quantifying iron levels in vivo using QSM could possibly provide useful biomarkers for diagnosing AD.

In a relatively small cohort (8 controls and 8 AD patients), Acosta-Cabronero et al. investigated susceptibility values using both region-based and whole-brain analysis approaches. Abnormalities of susceptibility values were found in several gray and white matter regions. The most interesting finding was in the putamen, where a higher susceptibility value was found in AD patients compared with controls (104). Promising results were reported in another study of 6 AD patients and 10 controls. The susceptibility of gray matter was found to be higher in Aβ PET-positive AD patients compared with Aβ-negative healthy controls (105).

Inconsistent results have also been reported by recent studies of patients at early stages of the disease. A study of 18 mild cognitive impairment (MCI) patients and 22 healthy controls showed no significant difference in the basal ganglia and cortical gray matter between groups, suggesting that magnetic susceptibility may not be sufficient to serve as a biomarker for diagnosis at early stages of disease initiation (106). Another study of MCI patients and healthy elderly individuals investigated the relationship between magnetic susceptibility and Aβ measured by Pittsburgh compound B PET. Although no correlation was found for healthy controls, strong positive correlations were observed in the caudate nucleus and frontal, temporal, parietal, and occipital lobes for MCI patients. These findings suggested that cerebral iron accumulation might reflect Aβ-associated brain dysfunction (107). One possible explanation for the discrepancy between these 2 studies could be the high heterogeneity of the MCI group.

To date, QSM studies of AD are scarce, yet the results are encouraging. Further studies dedicated to relating susceptibility with other established biomarkers for diagnosing AD and especially MCI are needed to provide additional information for establishing the role of QSM for diagnosing and managing AD patients.

### Oxygenation

Oxygenation imaging could provide biomarkers for studying cerebral physiology and improving understanding of disorders in which the oxygen supply is disturbed, such as stroke, tumor, and Alzheimer disease. In vessel segments that can be approximated as an infinite cylinder, the susceptibility difference between vein and tissue follows ΔX_vein–tissue_ = ΔX_do_ × Hct × OEF, where ΔX_do_ = 0.18 ppm is the susceptibility difference per unit hematocrit (Hct) between fully deoxygenated blood and fully oxygenated blood. Oxygen extraction fraction (OEF) can be calculated by measuring the susceptibility difference ΔX_vein–tissue_. According to the Fick principle, the cerebral metabolic rate for oxygen (CMRO_2_) can be expressed as CMRO_2_ = OEF × CBF × *C*_*a*_, where the carrying capacity of oxygen molecules per volume of blood (*C*_*a*_) is a typical constant. By measuring cerebral blood flow (CBF) using other MRI protocols such as arterial spin labeling, local CMRO_2_ could be also estimated.

Several QSM-based studies sought to measure OEF and CMRO_2_ and to compare the measurements to previously published results based on other MRI methods and PET imaging. In 12 healthy volunteers, Fan et al. reported a mean venous oxygen saturation of 59.7 ± 2.4% and a mean CMRO_2_ of 151 ± 15 μmol/100 g/min using the QSM-based method (108). Similarly assuming constant arterial oxygenation saturation level and total hemoglobin concentration, Zhang et al. generated quantitative maps of CMRO_2_ and OEF before and after caffeine vasoconstriction in 13 healthy volunteers. The reported CMRO_2_ of 153 ± 26.4 μmol/100 g/min agreed well with previous MRI and PET literature (109). In 10 healthy individuals, Fan et al. also measured a strong reduction of local venous OEF during hypercapnia relative to baseline. For instance, OEF decreased by 40% in the straight sinus and in the internal cerebral veins that drain the deep gray matter (110). In MS patients, Fan et al. also observed a 3.4% absolute reduction of mean cortical OEF in MS relative to healthy controls. A weak correlation between OEF and cortical thickness was observed. Interestingly, OEF strongly correlated with cognitive performance, particularly information-processing speed. A trend of progressive decrease in OEF with MS disease type was also reported (111).

More recent studies of patient populations have focused on comparing QSM-derived OEF measurements directly with PET-based OEF measurements. In 27 patients with steno-occlusive cerebrovascular diseases, Uwano et al. reported a strong correlation between the OEF ratio on the QSM-OEF maps and that on the PET-OEF maps (*r* = 0.89, *P* < .001) (112). In another study of 26 patients with chronic cerebral ischemia, Kudo et al. reported a moderate correlation between QSM-OEF and PET-OEF measured by the gold standard ^15^O PET (*r* = 0.60, *P* = .001) (113). These works demonstrated that QSM-based noninvasive measurements of OEF and CMRO_2_ can provide information regarding cerebral physiological changes and raise the prospect of QSM as an alternative to ^15^O_2_ PET for accessing patients with disruption in cerebral metabolism.

### Practical Matters of Clinical Translation

It is relatively straightforward to collect QSM data on a typical clinical MRI scanner because QSM uses the widely available 2D or 3D GRE sequence. In fact, many clinical protocols are already collecting 2D or 3D GRE data to obtain T2* or susceptibility weighting. A typical protocol for neural applications at 3T would be able to achieve a whole-brain coverage at an approximately 0.8-mm in-plane resolution and with a slice thickness approximately 2 mm in approximately 6 minutes of scan time. Faster scanning can be achieved with echo-planar imaging (EPI), spiral trajectories or the recently proposed Wave-CAIPI technique (114–117). Although 2D EPI is generally available, the other faster sequences are not yet widely available on clinical scanners. As susceptibility contrast and signal-to-noise ratio improves with field strength, it is generally beneficial to use a higher field strength when possible. Higher field strengths shorten T2*, allowing for shorter TEs and TRs and thus faster scans. For a given retention time, it is also beneficial to collect as many echoes as possible for improved efficiency and signal-to-noise ratio through multiecho averaging.

Currently, the main hurdle for broadly translating QSM into the clinics is that MRI vendors have yet to implement the necessary algorithms on their commercial scanners. First, most scanners do not store the phase images by default. Some susceptibility-weighted imaging protocols output phase images that are high-pass filtered, which removes much of the useful phase information. Some scanners produce phase images that contain discontinuities of singularity points or “open fringe lines” that are usually caused by an incorrect combination of images produced by multichannel coils. Given that the manufacturers are still working out their preferred ways to generate phase images, it still makes sense currently to store the unprocessed complex images of each coil and process them offline with in-house written or publically available software. If this is impractical for reasons such as increased storage space, it should be ensured at a minimum that the phase images generated by the scanner are not filtered improperly. Second, MRI vendors have not implemented QSM algorithms to solve the phase-to-susceptibility inverse problem. However, there is shareware (eg, STI Suite from Duke University) available for research purposes (13). Nevertheless, to broadly evaluate and apply QSM in clinical radiology would require the scanners to generate QSM maps automatically.

Although QSM of the brain has been most widely evaluated and is most readily translatable to the clinics, QSM of the body remains to be fully developed and optimized. The main challenge of body QSM is dealing with motion and water fat separation. However, early reports have shown promises in the kidney (23, 38), liver (24), heart (22), and cartilage (118, 119).

## Conclusions

QSM has revealed extensive variations of magnetic susceptibility among biological tissues and between healthy and diseased tissues. Studies have shown that these variations are most often caused by their unique composition of molecules with distinctive magnetic properties and their microscopic tissue organization. Normal physiologic and abnormal disease processes can cause changes in the molecular and cellular level, resulting in measurable changes in magnetic susceptibility. QSM is thus becoming a valuable MRI tool for quantitatively assessing tissue property.
